# Role of Preoperative Thyroid-Stimulating Hormone Levels in the Prediction of Thyroid Hormone Replacement after Hemithyroidectomy

**DOI:** 10.1055/s-0045-1801852

**Published:** 2025-04-15

**Authors:** Ramona Paula Fernandes Reckziegel, Lenara Golbert, Erika Laurini de Souza Meyer

**Affiliations:** 1 Endocrinology Service, Irmandade da Santa Casa de Misericórdia de Porto Alegre, Universidade Federal de Ciências da Saúde de Porto Alegre, RS, Brazil

**Keywords:** thyroidectomy, hypothyroidism, thyroid hormones

## Abstract

**Introduction** Hemithyroidectomy is performed for the treatment of symptomatic unilateral benign nodules, cytologically indeterminate nodules, and some cases of well-differentiated thyroid cancer.

**Objective** To evaluate the frequency of postlobectomy thyroid hormone replacement (THR), and to analyze the clinical-pathological factors predicting L-thyroxine (T4) use in patients undergoing hemithyroidectomy.

**Methods** We conducted an observational, retrospective study in which clinical, biochemical, and anatomopathological parameters were analyzed and correlated with the need for THR after thyroid lobectomy.

**Results** The frequency of postoperative THR was 63%. The preoperative thyroid-stimulating hormone (TSH) level was an important predictor of postoperative THR. When stratifying preoperative TSH levels, the frequencies of T4 replacement in each TSH quartile varied, being more frequent with increasing presurgical TSH levels ( *p* = 0.029). The preoperative cutoff that maximized sensitivity and specificity for the development of hypothyroidism was 1.21 μIU/mL.

**Conclusion** Our results demonstrated a significant frequency of postlobectomy THR. Higher preoperative TSH is a strong risk factor for postsurgical hypothyroidism, and even lower preoperative levels within the normal references do not exclude the risk of thyroid hormone use after thyroid lobectomy.

## Introduction

Hemithyroidectomy, or thyroid lobectomy, is indicated for benign and malignant thyroid disease, including cytologically indeterminate nodule, atypia of undetermined significance/follicular lesion of undetermined significance (AUS/FLUS), follicular/Hürthle cell neoplasm (FN/HCN), and cases in which there is a suspicious of malignancy. 1 2 In contrast to total thyroidectomy, this procedure avoids the occurrence of hypoparathyroidism and bilateral recurrent laryngeal nerve injury. It also correlates with a lower risk of postoperative neck hematoma. 2 3 Likewise, thyroid lobectomy provides the possibility of patients not requiring lifelong hormone replacement therapy. 1 2 Thus, the occurrence of hypothyroidism after lobectomy is an important factor in decision-making for the individual patient when deciding which surgical procedure will be performed. 2

The risk of hypothyroidism after hemithyroidectomy varies greatly between studies, ranging from 22% to 55.8%. 4 5 6 7 8 9 10 This discrepancy may be caused by differences in the studied populations, criteria for initiation of L-thyroxine (T4) therapy, and surgical techniques. 6

Consistently reported data demonstrated that the anatomopathological presence of chronic lymphocytic thyroiditis, higher than normal range of preoperative thyroid-stimulating hormone (TSH) levels, and positive anti-thyroid peroxidase antibodies (TPOAb) in the blood are risk factors for the development of postoperative hypothyroidism. 6 Nevertheless, the predictive role of other clinical or pathological factors remains unclear. 6

In fact, preoperative TSH levels are one of the most frequently reported risk factors among several studies. 4 5 6 7 8 9 10 11 12 A higher than normal range usually indicates deficiency of reserve thyroid function. 10 11 However, the TSH levels that predict postsurgical hypothyroidism are not well established, given that different cutoff points were used in the studies.

The present study aims to determine the predictive clinical-pathological factors for thyroid hormone replacement (THR) in patients undergoing hemithyroidectomy. Additionally, we also aim to assess the role of single serum TSH measurement before hemithyroidectomy on subsequent postoperative thyroid status.

## Methods

### Study Population and Design

We performed a retrospective study of patients undergoing hemithyroidectomy from January 2014 to March 2021 at our institution, a tertiary healthcare center, a teaching hospital in Southern Brazil. Thyroid lobectomy, or hemithyroidectomy, was defined as total removal of the unilateral thyroid lobe with or without isthmusectomy. The study received approval of the local ethics committee (CAAE 46964021.2.0000.5335). Patients with missing histopathological data, incomplete clinical history data, and pregnant women were excluded from the analyses.

### Characterization of Thyroid Function Status in Follow-up Assessments after Hemithyroidectomy

Euthyroidism was characterized as normal levels of serum TSH and free L-Thyroxine (FT4). Subclinical hypothyroidism was defined as a mild elevation of TSH levels beyond the upper limit of the reference range, and FT4 as levels within the normal reference range. Overt hypothyroidism was characterized as an increase in TSH levels above the reference range and a decrease in FT4 below the reference range.

At our institution, the reference range of TSH is 0.55 to 4.78 μUI/mL. The FT4 values changed over time, considered normal from 0.7 to 2.0 ng/mL between 2014 and 2016, and 0.89 to 1.76 ng/mL, since 2017. Patients were defined to be on THR if any dose of synthetic L-Thyroxine (T4) therapy was prescribed during follow-up.

### Assessment Parameters

We analyze the presurgical patient characteristics, including age, surgery duration, hospitalization time, sex, body mass index (BMI), TSH, free and total T4 levels, presence of TPOAb, preoperative thyroid ultrasound, final histological results after surgery, and coexistence of chronic lymphocytic thyroiditis (Hashimoto's thyroiditis).

The data of each TPOAb assay were recorded as “positive” or “negative” based on a titer above or below the cutoff point. A diagnosis of Hashimoto's thyroiditis was determined only on the final histological results.

### Statistical Analysis

Clinical and laboratory data are demonstrated as the mean ± standard deviation (SD) or median and percentiles 25 to 75 (P25–75) for continuous variables and absolute numbers and percentages for categorical variables. To evaluate the association between categorical variables, the chi-squared test was used. Receiver operating characteristic (ROC) curve analysis was applied to identify the optimum cutoff value of preoperative serum TSH for predicting the probability of postoperative TH replacement. A binomial logistic regression was performed to evaluate the impact of each variable at risk of postoperative TH replacement. Variables associated with need for TH therapy, at the significance level of *p* < 0.20, in the univariate analysis were included in the multivariate analysis. The odds ratios (OR) and 95% confidence intervals (95%CIs) were calculated.

The disease-free survival curve (time until initiation of T4 therapy) was plotted using Kaplan-Meier method and the log-rank test was used to determine their significance. Data analysis was performed using IBM SPSS Statistics for Windows (IBM Corp., Armonk, NY, United States) software, version 25.0. Values of *p* < 0.05 were considered statistically significant.

## Results

### Preoperative Characteristics of Patients

In the present study, a total of 73 patients underwent hemithyroidectomy. Clinical and laboratory characteristics of the patients are shown in Table 1 . The mean age at surgery was 48.2 ± 16.5 years. Furthermore, 62 patients (84.9%) were women, and 58 (84.1%) were in euthyroidism. The indication for the procedure was due to compressive symptoms in 55 patients (75.3%), followed by indeterminate FNA or suggestive of malignancy in 9 (12.3%), esthetics in 8 (9%) and autonomous nodule with hyperthyroidism in 4 cases (5.5%). Based on the Bethesda classification, among the 9 cases that had an exclusive indication due to FNA, 4 were III, 4 were IV, and one was VI. Benign final histopathology was present in 91.8% of the patients.

**Table 1:** Presurgical characteristics of patients underwent lobectomy

Age (years) – mean ± SD	48.2 ± 16.5
BMI (kg/m ^2^ ) – mean ± SD	28.4 ± 6.1
Sex – n (%)	
Female	62 (84.9)
Thyroid US – n (%)	
Heterogeneous parenchyma	30 (48.4)
Homogeneous parenchyma	32 (51.6)
Previous thyroid status – n (%)	
Hypothyroidism	4 (5.8)
Hyperthyroidism	7 (10.1)
Euthyroidism	58 (84.1)
Surgical indication – n (%)	
FNA suggestive of malignancy	9 (12.3)
Compressive symptoms	55 (75.3)
Hyperthyroidism	4 (5.5)
Esthetics	8 (11.0)
Final histology – n (%)	
Benign	67 (91.8)
Malignant	6 (8.2)
Presence of thyroiditis on the anatomopathological examination – n (%)	6 (8.2)
TPOAb – n (%)	
Positive	6 (17.6)
Negative	28 (82.4)
Preoperative TSH –median (P25–75)	1.3 (0.74–2.0)
Preoperative FT4–median (P25–75)	1.1 (0.98–1.3)
Preoperative T4T – median (P25–75)	9.4 (8.2–11.7)
Total follow-up time (months) –median (P25–75)	8.5 (4–13)

**Abbreviations:** BMI, body mass index; FNA, fine needle aspiration; FT4, free thyroxine; P25, percentile 25; P75, percentile 75; SD, standard deviation; T4T, total thyroxine; TPOAb, anti-thyroid peroxidase antibodies; TSH, thyroid-stimulating hormone; US, ultrasonography.

### Frequency and Risk Factors for Thyroid Hormone Supplementation Following Hemithyroidectomy

After a median follow-up time of 8.5 (4–13) months, 29 of 46 patients who were followed up after surgery required TH replacement; thus, the incidence of T4 use following thyroid lobectomy was 63%. While 26 patients (89%) developed hypothyroidism within 6 months after hemithyroidectomy. The median interval from surgery to the initiation of T4 therapy was 6 (3.2–8.8) months.

The differences between the characteristics of patients with and without TH replacement after surgery is shown in Table 2 . There were no association in age, BMI, sex, sonographic echotexture of the thyroid, previous thyroid dysfunction, surgical indication, side of hemithyroidectomy, malignant anatomopathology, thyroiditis, TPOAb positivity, or preoperative FT4 levels between those who did and did not require thyroid hormone treatment. TSH was the only clinical parameter associated with the need for postoperative T4 therapy. Overall, those who required thyroid hormone had a higher preoperative TSH (median: 1.6 [1.0–2.7] μUI/mL) than those who did not (median: 0.8 [0.5–1.4] μUI/mL) ( *p* = 0.015).

**Table 2:** Association between clinicopathological factors and thyroid hormone replacement (THR) after thyroid lobectomy

	**THR** **(n = 29)**	**Non-THR** **(n = 17)**	***p*** **-value**
Age (years) – mean ± SD	46.9 ± 15.9	49.2 ± 11.7	0.601
BMI (kg/m ^2^ ) – mean ± SD	29.5 ± 5.9	26.7 ± 6.1	0.167
Female sex – n (%)	25 (86.2)	15 (88.2)	1.000
Thyroid US – n (%)			0.183
Heterogeneous parenchyma	16 (61.5)	6 (40.0)	
Homogeneous parenchyma	10 (38.5)	9 (60.0)	
Previous thyroid status – n (%)			0.447
Hypothyroidism	2 (6.9)	0 (0,0)	
Hyperthyroidism	3 (10.3)	3 (17.6)	
Euthyroidism	24 (82.8)	14 (82.4)	
Surgical indication – n (%)			
FNA suggestive of malignancy	4 (13.8)	1 (5.9)	0.637
Compressive symptoms	22 (75.9)	13 (76.5)	1.000
Hyperthyroidism	2 (6.9)	2 (11.8)	0.619
Esthetics	3 (10.3)	2 (11.8)	1.000
Laterality – n (%)			0.148
Right	19 (70,4)	9 (47,4)	
Left	7 (25.9)	10 (52.6)	
Isthmectomy	1 (3.7)	0 (0.0)	
Malignant on the anatomopathological examination – n (%)	4 (13.8)	2 (11.8)	1.000
Presence of thyroiditis on the anatomopathological examination – n (%)	5 (17.2)	0 (0.0)	0.142
TPOAb, n (%)			0.622
Positive	4 (22.2)	1 (8.3)	
Negative	14 (77.8)	11 (91.7)	
Preoperative TSH, median (P25–75)	1.6 (1–2.7)	0.8 (0.5–1.4)	**0.015**
Preoperative FT4, median (P25–75)	1.1 (1–1.2)	1.2 (1–1.2)	0.535
Total follow-up time (months), median (P25–75)	10.0 (5–13)	6.0 (3–12)	0.411

**Abbreviations:** BMI, body mass index; FNA, fine needle aspiration; FT4, free thyroxine; P25, percentile 25; P75, percentile 75; SD, standard deviation; TPOAb, anti-thyroid peroxidase antibodies; TSH, thyroid-stimulating hormone; US, ultrasonography.

### Predictive of Preoperative TSH Levels

**Fig. 1:**
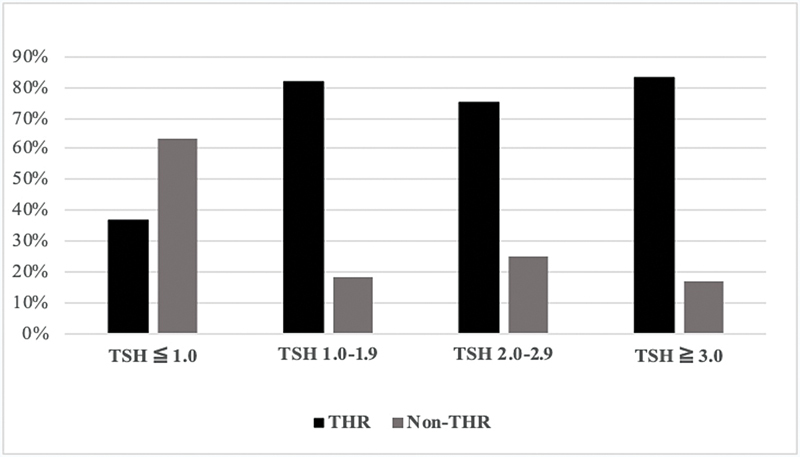
The percentage of patients with and without THR (Non-THR), according to preoperative TSH. **Abbreviations:** THR, thyroid hormone replacement; TSH, thyroid-stimulating hormone.

In univariate analysis, the only factor significantly correlated with TH replacement was preoperative TSH serum (OR: 2.43; 95%CI: 1.06–5.57; *p* = 0.035). The risk of TH replacement increases by 2.43 times, as preoperative TSH levels increase by 1 μUI/mL. In the adjusted multivariate analysis for hospitalization time and laterality of the procedure, which included variables at the significance level of *p* < 0.20, presurgical TSH was an independent predictor of postoperative T4 supplementation (OR: 2.34; 95%CI: 1.02–5.37; *p* = 0.046). To establish the role of TSH levels in postsurgical TH replacement the TSH were analyzed into the following groups: ≤ 1.0, 1.0 to 1.9, 2.0 to 2.9, and ≥ 3.0 μUI/mL. The percentage of patients with and without TH replacement in each group is shown in Figure 1 .

**Fig. 2:**
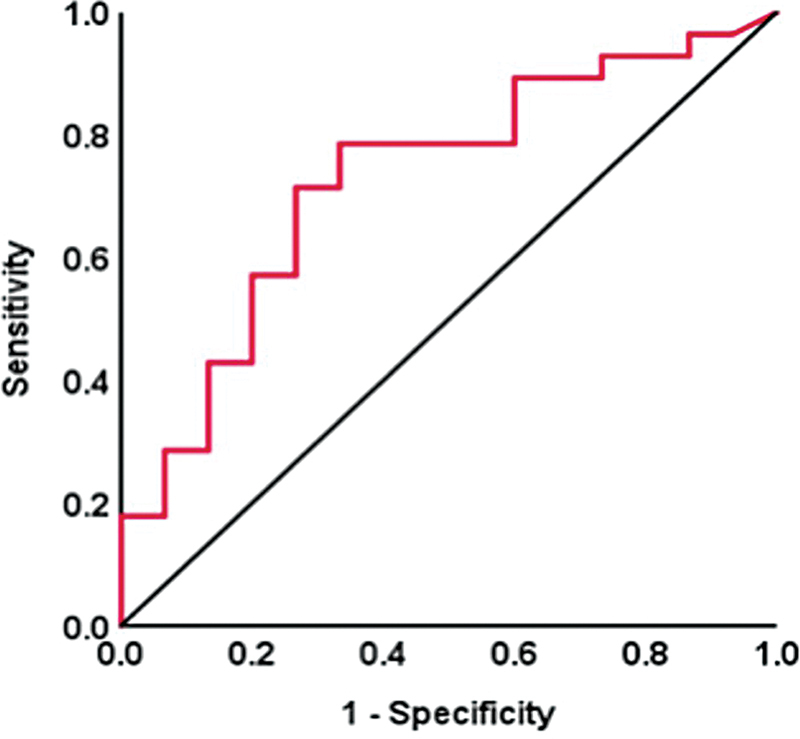
The ROC curve of the TSH with cutoff at 1.21μIU/mL. The AUC is 0.727, 95% CI is 0.568 to 0.887, and *p* is 0.015. **Abbreviations:** AUC, area under curve; ROC, receiver operating characteristic; TSH, thyroid-stimulating hormone.

TH supplementation was more frequent in those with increasing presurgical TSH levels ( *p* = 0.029). Given that the TSH level was defined as the most important risk factor for TH supplementation, we determine that a specific preoperative TSH cutoff level is greater than or equal to 1.21 μIU/mL. The area under the ROC curve (AUC) is 0.727 (95%CI: 0.568–0.887; sensitivity, 71.4%; specificity, 73.3%; *p* = 0.015), as shown in Figure 2 .

**Fig. 3:**
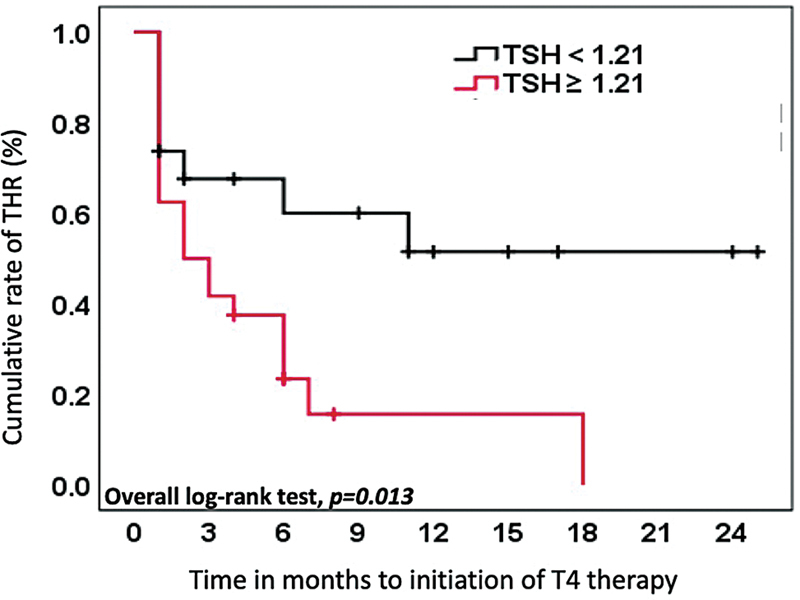
Cumulative rate of THR over time according to cutoff TSH value. **Abbreviations:** THR, thyroid hormone replacement; TSH, thyroid-stimulating hormone.

The risk of TH supplementation after hemithyroidectomy increases 2.5 times in patients with preoperative TSH greater than or equal to 1.21 μIU/mL compared to those below 1.21 μIU/mL (OR: 2.53; 95%CI: 1.09–5.88; *p* = 0.031). Additionally, the median time to initiation of T4 therapy after hemithyroidectomy was 14.6 (9.3–20) months in patients with preoperative TSH below 1.21 μIU/mL, and 5.2 (2.6–7.9) months in patients with preoperative TSH greater than or equal to 1.21 μIU/mL ( *p* = 0.013), as shown in Figure 3 .

## Discussion

Our results confirm that preoperative TSH levels are an independent factor in predicting THR after lobectomy. Interestingly, even patients with TSH levels within the lower normal range might need levothyroxine supplementation. A presurgical TSH level ≥ 1.21 μIU/mL was correlated with the risk of post hemithyroidectomy T4 replacement.

The other variables (age at operation, sex, BMI, heterogeneity in preoperative ultrasound, positive TPOAb, lymphocytic thyroiditis at final histology, and presurgical T4 levels) are not associated with TH replacement at follow-up.

Our data shows that the overall incidence of TH supplementation following thyroid lobectomy is of 63%, which is relatively higher than that reported in previous studies (5.6–55.8%). 6 8 The higher incidence observed in our cohort may be due to the nonstandardized levothyroxine supplementation protocol by different assistants. In fact, some studies have demonstrated that postoperative hypothyroidism, including subclinical, can occur in 64% of patients undergoing lobectomy. 13 However, without immediate T4 replacement, approximately 68% of patients spontaneously recover thyroid function. 13 In fact, after hemithyroidectomy, most patients will remain in euthyroidism and immediate start of T4 may lead to TSH suppression. 14

Some studies have demonstrated that, in patients with benign pathology, the rate of T4 use was lower compared to those with malignancy. 4 5 In order to reach the optimal TSH goal (< 2.0 μIU/mL) to be compliant with the American Thyroid Association's recommendation for patients with differentiated thyroid carcinoma, 50 to 73% of patients with malignant histological results need postoperative levothyroxine. 1 12 15 Nonetheless, 92% of the histological results in our study were benign, which indicates that even in benign pathologies the need for TH replacement is significant. Additionally, a recent retrospective study reported a higher rate of T4 initiation after lobectomy for benign disease than prior studies. 16

Some clinical and pathological factors may influence the need for T4 supplementation following hemithyroidectomy. Preoperative TSH level, positive TPOAb, and chronic lymphocytic thyroiditis have been associated with postoperative hypothyroidism. 6 17 Similar to previous studies, we have demonstrated that presurgical serum TSH levels are an independent risk factor for postoperative hypothyroidism, with approximately double the risk of T4 replacement for every unit of TSH increase over 1 μIU/ml. 8 11 18 In our sample, preoperative TSH remained an important factor despite the lack of association with the other variables.

Defining a specific presurgical TSH cutoff level instead of considering only the highest levels can be very useful in preoperative patient counseling regarding the risks of T4 replacement after surgery. Nonetheless, different cutoff levels of TSH have been applied in the studies, some being established by the authors. We established the specific cutoff level associated with the risk of THR after lobectomy by using ROC curves. In our study, presurgical TSH levels of at least 1.21μIU/mL were associated with hormone treatment following lobectomy, with about 2.5-fold higher risks. Interestingly, our cutoff was lower compared to previous studies, most of which preferred 2.0 μIU/mL. A presurgical TSH level greater than 2.0 μIU/mL was associated with a risk ratio of 2.955 (95%CI: 2.399–3.640; *p* = 0.000) for hypothyroidism after lobectomy compared to those under 2.0 μIU/mL. 17

In recent studies, preoperative serum TSH > 2.172 μIU/mL was demonstrated as an independent risk factor for T4 replacement after surgery (OR = 8.02; 95%CI: 4.87–13.20; *p* < 0.001). 19 The cutoff values defined in other studies ranged from 1.4 to 2.5 mIU/L. 10 11 12 13 20

Similar to previous studies, most patients of our cohort started replacement with T4 early in the postsurgical period; almost 70% of them within 3 months, and about 90% within 6 months. 8 19 Interestingly, the time to initiation of T4 therapy in patients with preoperative TSH < 1.21mIU/L was longer, demonstrating that late onset postoperative hypothyroidism of 1-year or more after lobectomy can happen, indicating a possible longer follow-up for thyroid function evaluation.

Several previous studies have demonstrated that higher presurgical TSH levels combined with lymphocytic infiltration of the thyroid tissue are associated with postoperative hypothyroidism. 4 6 8 17 However, we found that anatomopathological findings on thyroiditis was not significantly associated with the need for postoperative T4 supplementation. These results should be viewed with caution because of the small number of thyroiditis patients with histological description in our cohort. This may be related to the inconsistency in pathological reporting or due to selection bias recommending total thyroidectomy for patients with related clinical history or preoperative ultrasonography findings. Nonetheless, this factor can be reliably assessed after final histological evaluation and is not suitable for thyroid function prediction before lobectomy.

In the same way we did not find a difference in TPOAb levels between patients who required or not TH supplementation. Previous studies have shown that TPOAb-positive patients had a relevant risk (about 50%) of postsurgical hypothyroidism in comparison with negative ones. 6 On the other hand, this association was not confirmed in a recent meta-analysis. 17 Indeed, the preoperative measurement may be used as a simple tool to estimate the risk of hypothyroidism following surgery, but it is not universally recommended in preoperative evaluation of partial thyroidectomy patients.

There are several limitations to this study. It's a retrospective study performed at a single tertiary referral center. Our academic medical center, despite a high volume of thyroidectomies, still recommends lobectomy for a small number of patients; in addition, some patients had no postsurgical follow-up, which may overestimate the prevalence of TH replacement. Another possible limitation is the lack of standardization of THR, which prevented us from assessing the proportion of patients with transient hypothyroidism. On the other hand, the data reflect the real-life of clinical practice.

## Conclusion

In conclusion, thyroid hormone supplementation is common after lobectomy. In our cohort, many cases had benign pathology, patients with preoperative TSH level > 1.21μIU/mL are at risk of levothyroxine supplementation. Prospective studies with a larger number of participants applying the same follow-up protocol are important to clarify the role of other clinical factors in risk of THR following hemithyroidectomy.
